# The Care of the Teeth

**Published:** 1904-04

**Authors:** 


### THE CARE OF THE TEETH.

The secret of the preservation of the teeth is absolute cleanliness. The first rule is to cleanse the teeth thoroughly after eating, no matter how times a day. A tooth wash should be for the most part of soap in some form, since soap is both cleansing and alkaline. The next matter of importance is the tooth brush. It is well to have the two kinds, one in which the bristles are even and the other in which the bristles have been cut across so as to be in points. Each tooth must be thought of as something to be brushed in its entirety, front, back, sides, and the junction with the gum. Once a month is none too often to have a new brush. The teeth should be brushed with the hand of the opposite side; that is, the right hand for the teeth of the left side and the left hand for the right. After brushing the teeth an antiseptic mouth wash should be used. If the teeth become yellow or show discolored spots in spite of good care a little powdered pumice stone should be used.—From "The Fountain of Youth," in the May *Delineator*.

